# Energy poverty and the convergence hypothesis across EU member states

**DOI:** 10.1007/s12053-023-10113-9

**Published:** 2023-05-05

**Authors:** Athanasios Anastasiou, Eftychia Zaroutieri

**Affiliations:** Laboratory of Data Science and Digital Transformation, Department of Management Science and Technology, University of Peloponnese, Tripoli, Greece

**Keywords:** Convergence, Energy poverty, Log-*t* regression test, C50, Q43, E01

## Abstract

Energy poverty is an emerging issue towards global affairs. Currently, the development of energy-related policies is becoming essential, with regard to new societies, social inclusion and social rights. In this paper, we examine the dynamic patterns of energy poverty among 27 EU member states between 2005 and 2020. We use the log-*t* regression test to investigate the convergence hypothesis, and the P&S data-driven algorithm to detect potential convergence clubs. The empirical results of energy poverty indicators are mixed, and the convergence hypothesis of the states is rejected. Instead, convergence clubs are exhibited, implying that groups of countries converge to different steady states in the long run. In view of the convergence clubs, we suggest that the affordability of heating services is potentially explained by structural conditions of housing, climate conditions and energy costs. Besides, the adverse financial and social conditions for the European households have significantly triggered the arrears on utility bills. Moreover, a significant proportion of households do not have basic sanitation services.

## Introduction

International organizations and government agencies are deeply concerned about tackling energy issues as a pillar of achieving major development goals (Barnes et al., 2011; He et al., 2022). In the light of this consideration, the United Nations defined “Affordable, reliable, sustainable and modern energy for all” as an objective for the implementation of the 2030 Agenda. The European Pillar of Social Rights affirms that energy is an essential source which any household should be granted. Hence, energy poverty is a core issue in civil society and a priority that forms the pillar of multiple initiatives and policy implications, set by the European Commission and its stakeholders (Bouzarovski & Tirado Herrero, 2017). Besides, the energy sector undergoes rapid changes and challenges followed by the global concerns about climate change, shift in energy prices, social welfare and the sustainable development goals (González-Eguino, 2015).

Recently, an academic and policy knowledge has grown over energy poverty, arguing that the last is a social rather than an inherently environmental or economic issue, followed by global affairs (Samarakoon, 2019). Verily, vulnerable consumers where inevitably hit by the adverse effects of the outbreak of COVID-19 pandemic on the energy sector (Carfora et al., 2022; Hesselman et al., 2021). The rising energy prices accompanied by the pandemic made several people unable to pay their energy bills (Baker et al., 2021). As a result, final energy use was significantly reduced by the household sector and mainly by vulnerable groups such as lower-income households (Clark et al., 2021). Nevertheless, it is not only the health crisis that caused detrimental effects on energy security and conservation, but also energy geopolitics. Concurrently, Russia’s war of aggression against Ukraine and the immediate response of nations, i.e. the imposed sanctions to the country, intensified the existing energy crisis in the European Union (Siksnelyte-Butkiene, 2022). The burden of rising prices is heavily borne by households through the rising cost of fuels, which are essential for heating, cooling, transport and other daily needs, as well as via the decrease in disposable income, i.e. the higher share of energy costs on households’ expenditures (Biernat-Jarka et al., 2021). Nevertheless, cross-country differences are observed, implying that the effects of rising prices and other energy-related issues of individual welfare and prosperity are disturbed by country-specific characteristics (Carfora et al., 2022). Hence, energy-related issues of disposable income and the living standards of individuals should be considered both at regional and international levels.

Even though the academic community dives into the causes and effects of energy poverty, the dynamic behaviour of energy poverty across economies is ambiguous. Understanding the evolutionary patterns is essential in terms of policymaking and decision. We then throw that the investigation of energy poverty patterns among EU member states is a precondition for the European Commission to enact effective policy measures. The analysis of different aspects of energy poverty convergence, i.e. the exploitation of different indicators, is essential in the measurement of disparities. This contributes to the detection of convergence behaviour between members which is inherently affected by the definition; viz., a country may appear in one cluster when using one indicator, while it may be included in a different club when another indicator is considered. In all respects, potential convergence clubs provide an understanding of the evolution of energy poverty, given different aspects of the term. Besides, the existence of any clusters implies that energy poverty declines faster in some economies rather than the other ones. This, however, enables policymakers to design country-specific policy measures and initiatives rather than uniform policies which may be ineffective for some states.

In this framework, the purpose of this study is to investigate the dynamic patterns of energy poverty across 27 EU member states between 2005 and 2020. To be more precise, we exploit the log-*t* regression test, as proposed by Phillips and Sul (2009), and we examine the convergence hypothesis for the whole panel. Besides, we use the data-driven algorithm to detect potential convergence clubs of the EU economies. We are then concerned to answer the following research questions that currently originate from the energy poverty. Our research questions are as follows: Do the European economies converge to an equilibrium steady state? Given that this hypothesis is violated, do countries define potential convergence clubs? If we account for different aspects of energy poverty, in what level do the results vary?

## Literature review

### Energy poverty

The understanding of energy poverty requires a consistent and valid definition, which is innately misleading and ambiguous (Deller et al., 2021). Besides, an inaccurate definition of the term makes the identification of households who live under the risk of energy poverty difficult for academic community and policymakers (Pachauri & Rao, 2020). Moreover, both energy poverty and poverty are abstruse concepts, i.e. the first accounts for the energy-related aspect of the second (Halkos & Gkampoura, 2021a, 2021b). Nevertheless, an acknowledged definition of energy poverty relates to the inadequate access to energy services; viz., energy-poor are the individuals/households which are unable to afford basic needs in their homes, such as cooking, heating, cooling and lighting (Castaño-Rosa et al., 2019; Turai et al., 2021). Energy poverty is a multi-dimensional concept which is captured by different aspects. The European Commission argues that a single definition of energy poverty is inexistent, although accepting that it is kindly expressed by the inability to keep homes adequately warm (Thomson & Bouzarovski, 2020). Energy poverty is related to low income, high-energy prices and energy inefficiencies which are technically associated to infrastructures (Brucal & McCoy, 2021). Verily, this issue reflects the economic and social conditions within economies, i.e. it illustrates different aspects of poverty and welfare which are attributed to the distribution of energy (Karpinska & Śmiech, 2020). To that end, the concept is viewed over two perspectives, that is over a micro-survey and a macro-perspective (Rademaekers et al., 2016). Primary indicators are designed to capture energy poverty from the view of disposable income and spending for energy services, while secondary ones reflect multi-dimensions, i.e. indicators that reflect energy prices, housing infrastructure, building stock features and poverty levels.

From a different perspective, the access to energy services is the ability that an individual acquires, in terms of social integration (Bouzarovski et al., 2020; Hanke et al., 2021). This arises from the fact that the distribution of energy across households is uneven, implying that energy poverty can increase inequalities and threaten individual welfare and health conditions (Bouzarovski et al., 2014; Thomson & Bouzarovski, 2018). To be more precise, the inability of covering daily needs, e.g. cooking, cooling, heating and lighting, is evident in several households across economies. Approximately 8% of the European population is unable to keep their home adequately warm in 2020, while large disparities exist across the EU member countries. The difficulties of households to meet basic human needs generate barriers that burden well-being and human development.

In consequence, the European Commission is concerned about tackling energy poverty and adopts long-term schemes and initiatives to mitigate energy, social and economic disparities. The global 2008 financial recession and the outbreak of 2019 health crisis had a deep and long-lasting impact on social and economic inequalities among EU countries (Anastasiou, 2009; Arbolino & Di Caro, 2021; Baldwin & Di Mauro, 2020; Conte et al., 2020; Dayrit & Mendoza, 2020; Zervoyianni & Anastasiou, 2007, 2009; Zervoyianni et al., 2014). Recently, the European policy measures against poverty and income inequalities lie around the promotion of convergence to a minimum steady state. Howbeit, the understanding of disparities in economic and social sectors, followed by the aforementioned issues, is ambiguous. Precisely, the real gains from policy measures and initiatives vary across member states, despite the formation of a unified economic area (Bouzarovski & Tirado Herrero, 2017). From a holistic perspective, it is essential to understand how energy disparities and wider socioeconomic inequalities relate to each other, via the lens of differences in energy poverty outcomes.

### Convergence

The concept of convergence was originally introduced to explain the evolution of per capita income, i.e. economic disparities exhibiting from the integration to international trade activities, capital accumulation, labour and capital productivity (Solow, 1956). In broader terms, the catch-up effect implies that, as economies exhibit higher growth rates, the per capita income is gradually transferred from the developed to developing countries. The dynamic behaviour explains how capital is accumulated in poor countries, so that a steady state between wealth and poor economies is achieved (Barro, 1991; Barro et al., 1991; Barro & Martin, 1992). Early studies on convergence are grounded on economic growth, while recent work is delved into the fields of energy and environmental economics. Distinctive studies focus on energy efficiency (Cheng et al., 2020; Liu & Li, 2018; Markandya et al., 2004; Pan et al., 2015; Zhang et al., 2017), consumption (Akram et al., 2020; Kim, 2015; Mishra & Smyth, 2017; Pan & Maslyuk-Escobedo, 2019) and intensity (Markandya et al., 2006; Solarin, 2019; Szép, 2016; Zhang & Broadstock, 2016).

The literature on convergence consists of σ-convergence, β-convergence, stochastic convergence and club convergence. Precisely, σ-convergence captures the cross-sectional equilibrium over time (viz., the variation of per capita income between economies reduces) (Quah, 1996), while β-convergence is attributed to the increasing growth rates of less-developed economies. The last concept implies that a steady-state equilibrium is achieved when poorer nations exhibit rapid growth, which is mainly led by an increase in marginal productivity and diminishing returns to capital, leading to a long-run convergence (Horta & Camanho, 2015). To this end, β-convergence examines the relation between per capita income at initial stages and following growth rates of economies (Costantini and Lupi, 2005). If the correlation is significant and negative, β-convergence is achieved, while positive correlation implies a divergence behaviour of poor and wealth nations. Nevertheless, given the assumption of equal speeds of convergence, homogeneous technological progress and initial income between economies, the results of convergence or divergence between rich and poor economies may be biased and inconsistent (Durlauf, 2003; Phillips & Sul, 2003). Moreover, the unobserved heterogeneity between economies disturbs the empirical estimations, due to endogeneity and omitted variable issues.

Stochastic convergence then accounts for stationarity issues, i.e. convergence between states is achieved if the per capita income of a country relative to the benchmark country is an I(0) stationary process (Lee et al., 1997). In other terms, stochastic convergence suggests that long-run economic growth does not depend on idiosyncratic country-specific characteristics, rather these effects are temporary (Evans, 1998). Howbeit, stochastic convergence is sensitive to less effective univariate tests and misspecification errors, if time series issues are not considered, e.g. structural breaks or cyclical components (Bigerna et al., 2021; Carrion-i-Silvestre & German-Soto, 2009).

Given these shortcomings, multiple steady-state equilibrium points imply the existence of convergence groups. In view of this, the rejection of convergence hypothesis over a group of countries does not necessarily imply a divergence behaviour between subgroups of economic unities. In this view, Phillips and Sul (2007, 2009) have recently constructed a non-linear time-varying factor model, which accounts for individual and transitional heterogeneity of economies and is independent of trend stationarity assumptions of the series. A data-driven algorithm detects convergence clusters, even if the convergence hypothesis is rejected for the whole panel. The methodological approach of Phillips and Sul (2007, 2009) has many benefits over the conventional methods of convergence. First, there is no strict assumption of the stationarity of the variables and the common factors. Moreover, the method accounts for the changing behaviour of the economies, i.e. the transition heterogeneity or divergence. Third, the method has the capability to detect possible convergence clusters between heterogeneous economies, i.e. convergence paths where countries move towards a steady state in the long run (Saba & Ngepah, 2022a, 2022b). Fourth, the data-driven algorithm further enables the detection of club merging between the existing clusters, when the clustering procedure overestimates the real number of clusters (Saba & Ngepah, 2022a, 2022b).

Recently, studies have mainly focused on the impacts and causes of energy poverty (Ballesteros-Arjona et al., 2022; Neacsa et al., 2020; Papada & Kaliampakos, 2018; Primc et al., 2019), rather than the convergence hypothesis. Notwithstanding, the literature on energy poverty convergence is scare, while, to the best of our knowledge, two studies have investigated the hypothesis. Huang et al. (2022) exploit the method of moments quantile regression to examine whether 28 EU countries converge to an equilibrium steady state of energy poverty, while the empirical results confirm the hypothesis. Similarly, Salman et al. (2022) investigate the hypothesis, considering 146 economies. The empirical results of the Phillips and Sul algorithm suggest that the countries showcase divergence behaviour, but convergence clusters are existent. The rationale of this article is to examine the club convergence between 27 EU countries, considering three different aspects of energy poverty.

## Methodological approach

### Log-t regression test

A model of the panel data variable *X*_*it*_ is decomposed into a systematic component (*g*_*it*_) and a transitory component (*a*_*it*_) as follows: ${X}_{it}={\alpha }_{it}+{g}_{it}=\left(\frac{{\alpha }_{it}+{g}_{it}}{{\mu }_{t}}\right){\mu }_{t}={\delta }_{it}{\mu }_{t}$ for *i* = 1, 2,…, *N* economic unities which are observed between *t* = 1, 2,…, *T* periods. Then, ${\mu }_{t}$ is a single common component and ${\delta }_{it}$ is a systematic idiosyncratic element, i.e. a time-varying heterogenous component which accounts for the distance between ${X}_{it}$ and the common factor ${\mu }_{t}$. If ${\mu }_{t}$ is the common trend between individuals, then ${\delta }_{it}$ is a transition parameter as the share of ${X}_{it}$ in the common trend component. To estimate ${\delta }_{it}$, the authors construct a relative transition parameter as ${h}_{it}=\left(\frac{{X}_{it}}{\frac{1}{N}\sum_{i=1}^{N}{X}_{it}}\right)=\left(\frac{{\delta }_{it}}{\frac{1}{N}\sum_{i=1}^{N}{\delta }_{it}}\right)$. This parameter reflects the transitional behaviour of individual *i* with respect to the cross-sectional average at time *t*. The convergence hypothesis implies a common limit in the transition path between the economic unities so that ${h}_{it}\to 1$ for all *i* = 1, 2,…, *N*, as *t* $\to \infty$. If ${\delta }_{it}\to \delta$ as *t* $\to \infty$, then the cross-sectional variation should converge to zero as follows: ${H}_{it}=\frac{\sum_{i=1}^{N}{\left({h}_{it}-1\right)}^{2}}{N}\to 0.$ Phillips and Sul use the log-*t* regression test to construct the null hypothesis of convergence by technically using the following assumption for the idiosyncratic component ${\delta }_{it}$ where ${\delta }_{it}={\delta }_{i}+{\sigma }_{i}{\xi }_{it}{L(t)}^{-1}{t}^{-\alpha }$. Then, ${\delta }_{i}$ and ${\sigma }_{i}$ are constant; ${\xi }_{it}$ is i.i.d. with zero mean and variance 1, i.e. ${\xi }_{it}\sim (\mathrm{0,1})$; *L*(*t*) is a slowly varying function which eliminates the increase in the variance of log(*t*) where *L*(*t*)$\to \infty$ as *t* $\to \infty$; and $\alpha$ captures the decay rate, that is the rate of convergence. The null and the alternative hypotheses are then obtained as Ho: *δ*_*i*_ = *δ* and *α* ≥ 0 against H_1_: *δ*_*i*_ ≠ *δ* and *α* < 0. Under the null hypothesis, the whole panel convergence is achieved. To test the null hypothesis, the authors estimate the following regression model: $\mathrm{log}\left(\frac{{H}_{1}}{{H}_{t}}\right)-2\mathrm{log}(L(t))=\alpha +\beta \mathrm{log}(t)+{\varepsilon }_{t}$ for $t=$[*rT*], [*rT*] + 1,…, *T*, for all *r* > 0, where *rꞓ*(0.2,0.3). An optimal value of *r* is 0.2 for *T* ≥ 100. Then, the one-side *t* test of heteroscedasticity and autocorrelation is conducted for $\beta$ coefficient. The null hypothesis of convergence, i.e. Ho: *β* $\ge$ 0, is rejected if the *t*-statistic of the test is below − 1.65 as the critical value at the 5% level of significance, that is if ${t}_{\widehat{\beta }}=\frac{\widehat{\beta }-\beta }{\mathrm{se}(\widehat{\beta })}<-1.65$. Instead, the whole panel convergence is exhibited if ${t}_{\widehat{\beta }}>-1.65$.

### Club clustering algorithm

The rejection of the whole panel convergence does not strictly imply the existence of convergence clubs, i.e. subgroups of countries that converge to local steady states. Phillips and Sul (2007) hence developed an algorithm that explores potential clubs as follows:

1. Sort the individuals by descending order regarding the sample mean of the last period.
2. Find *k*, 2 ≤ *k* ≤ *N*: For each *k*, *k* + 1, *k* + 2,…, *k* + *j* individuals which are iteratively incorporated to a core group, conduct the log-*t* regression test until ${t}_{\widehat{\beta }}>-1.65$. If no *k* satisfies the convergence hypothesis, then no convergence subgroups are exhibited. Otherwise, find *k* such that *k* = arg max*k*{*tk*} subject to min{*tk*} >  − 1.65. The core group *G*_k*_ is then defined.
3. Initial club membership: For every individual which is not included in the core group, add one at a time to the core group and conduct the log-*t* regression test, until any individuals are available. If ${t}_{\widehat{\beta }}>-1.65$, then the individual is included in the initial core group *G*_k*_.
4. Recursion and stopping rule: The remaining individuals define a new subgroup, where the log-*t* regression test is conducted. If the convergence hypothesis is achieved, then two final subgroups are extracted. Instead, if the convergence hypothesis fails, then steps 1–3 are repeated to detect the existence of further clubs.
5. Club merging: When the initial clusters are determined, then for each one pair, i.e. club 1 + 2, club 2 + 3, club *k* − 1 + *k*, the log-*t* regression test is conducted. If the convergence hypothesis is accomplished, then these clubs merge into a larger group. This procedure is repeated until no groups can be merged. The final classification is completed.

## Data description and analysis

To detect the convergence patterns of energy poverty across EU member counties, we employ a dataset from the EU Statistics on Income and Living Conditions (EU-SILC). Despite the prevailing term of energy poverty, a uniform definition is difficult to be deployed, implying that a fundamental understanding is essential (Day et al., 2016). Energy poverty is a multi-dimensional factor which is reached by several indicators and methods (Bollino & Botti, 2017). These indicators according to the Energy Poverty Observatory are classified into two aspects, i.e. primary and secondary indicators, as already mentioned in a previous section.

Primary indicators are designed to capture energy poverty from the view of disposable income and spending for energy services, while secondary ones reflect multi-dimensions, i.e. indicators that account for energy prices, housing infrastructure, building stock features and deprivation levels (Sy & Mokaddem, 2022). Precisely, the Energy Poverty Observatory (EPOV) classifies primary indicators in consensual-based and expenditure-based indicators, which originate from the Household Budget Survey (HBS) micro-data. Consensual-based indicators reflect households’ potential to meet basic human needs which are energy related, i.e. the accessibility of modern and conventional energy services that are essential for cooking, cooling, lighting or heating needs. EPOV’s primary indicators are calculated directly from the responses of individuals, i.e. the indicators are based on self-assessment. Instead, expenditure-based indicators capture the level of energy poverty based on the household income. The indicators account for the share of household’s expenditure on energy services to the total disposable income or total household expenditure, or the level of household’s energy expenditures with respect to the minimum level of basic energy services (Halkos & Gkampoura, 2021a, 2021b). Expenditure-based indicators are determined by certain thresholds which define a level of disposable income or energy expenditures, below or beyond which households are qualified as energy-poor (Romero et al., 2018; Sareen et al., 2020). The thresholds set a minimum or a maximum level of these aggregates, so that households should spend to access modern energy services and be fairly integrated in society (Belaïd, 2022).

Secondary indicators of energy poverty comprise a range of variables which are extracted from the EU-SILC survey and the Building Stock Observatory (BSO). Even though these metrics are supporting indicators which can enrich the understanding of energy poverty outcomes, they are not sufficient for the measurement of energy poverty (Rademaekers et al., 2016). The secondary indicators are based on multiple areas, i.e. demographic factors (household size, tenure status, location), energy demand and supply (prices, tariffs), income (households in material deprivation or at risk of poverty), building efficiency (quality of building, tenure status, dwelling type) and policy intervention–based factors (social income support).

Presently, expenditure-based data coverage on energy poverty is limited; hence, HBS-based indicators are currently unavailable. Accordingly, we have access solely to consensual-based data. Hence, for our analysis, we employ a dataset with consensual-based and secondary indicators. The first primary indicator of energy poverty, from the Statistics on Income and Living Conditions (SILC) survey, pertains to the affordability of energy prices, that is, the population unable to keep their home adequately warm by poverty status as a share of the total population and expressed in percentage (EP1). Particularly, the indicator is retrieved from the “Environment and Energy” database for the “Sustainable Development indicators Goal 7”. We further exploit a different consensual-based indicator for the arrears of utility bills, i.e. the population unable to afford utility bills for the main dwelling on time, due to financial distress, as a share of the total population in percentage terms (EP2). On the other hand, we are interested in focusing on a different aspect of energy poverty that captures income conditions in terms of the affordability of certain commodities or services. In this view, we exploit a secondary indicator that embodies poverty and health risks, namely the material deprivation for the “Housing” dimension. The European Statistical Authority (Eurostat) has recently proposed the material deprivation rate as a measure of households’ ability to afford necessary or desirable items, in the matter of human well-being. Material deprivation is a newly introduced measure, which is designed to capture material living conditions of households and individuals. Withal, material deprivation rate is not a unique indicator, but it consists of several statistical indicators, each one of them corresponding to a different dimension. These indicators will potentially provide useful information on monetary poverty, which, in turn, avail policy coordination and progress monitoring for social exclusion and deprivation. Precisely, the indicators are intended to acquire information on different aspects of monetary poverty determinants, i.e. each item corresponds to an overall dimension of lifestyle deprivation relative to economic strain, durables and housing. For the aim of the study, we use material deprivation rate for the housing dimension from the SILC survey, for the households whose income is below 60% of median income. In this instance, we exploit < 0.6 M threshold, rather than > 0.6 M or total, to follow up on lower- and middle-income households in relation to the risk of poverty threshold. The material deprivation for the housing dimension corresponds to the share of individuals who are deprived in terms of materials, i.e. they reside in distressed dwellings. Eurostat defines materially deprived for housing those persons who have buildings where they suffer from at least one of the following items: (a) leaking roof, damp walls/floors/foundation or rot in window frames; (b) accommodation too dark; (c) no bath/shower; and (d) no indoor flushing toilet for sole use of the household. The indicator is calculated over the number of items, i.e. population who suffer from 0 item, 1 item, 2 items, 3 items or 4 items. However, we use the material deprivation of individuals who suffer from 3 out of the 4 items (EP3). Eventually, we collect data for 27 EU countries which are observed between 2005 and 2020 and the sample consists of 432 observations.

Table 1 represents the descriptive statistics of the energy poverty indicators. The distribution of EP indicators is positively asymmetrical, implying extreme values with respect to most frequent ones. Nevertheless, the log-*t* regression test and the club clustering algorithms are not sensitive to extreme values, due to the smoothing and standardization processes. The sample mean of the share of population who are unable to keep their home adequately warm is 11.5%, and the standard deviation is 12.1%, which justifies the potential asymmetries. Besides, the difference between the medians and the third quartiles (Q3) explicates the asymmetries which are either attributed to the disparities across members or the deviations within members over time.

**Table 1:** Descriptive statistics of EP indicators

	EP1 (%)	EP2 (%)	EP3 (%)
Mean	11.5	10.4	3.5
SD	12.1	8.3	5.5
Q1	3.1	4.5	0.2
Median	6.5	7.5	0.8
Q3	15.7	13.9	3.7
Min	0.3	1.1	0.0
Max	69.5	42.2	32.4
Skewness	2.05	1.43	1.98
Kurtosis	8.32	4.56	6.59

EP1, population unable to keep their home adequately warm; EP2, arrears of utility bills; EP3, material deprivation for the housing dimension

**Fig. 1:**
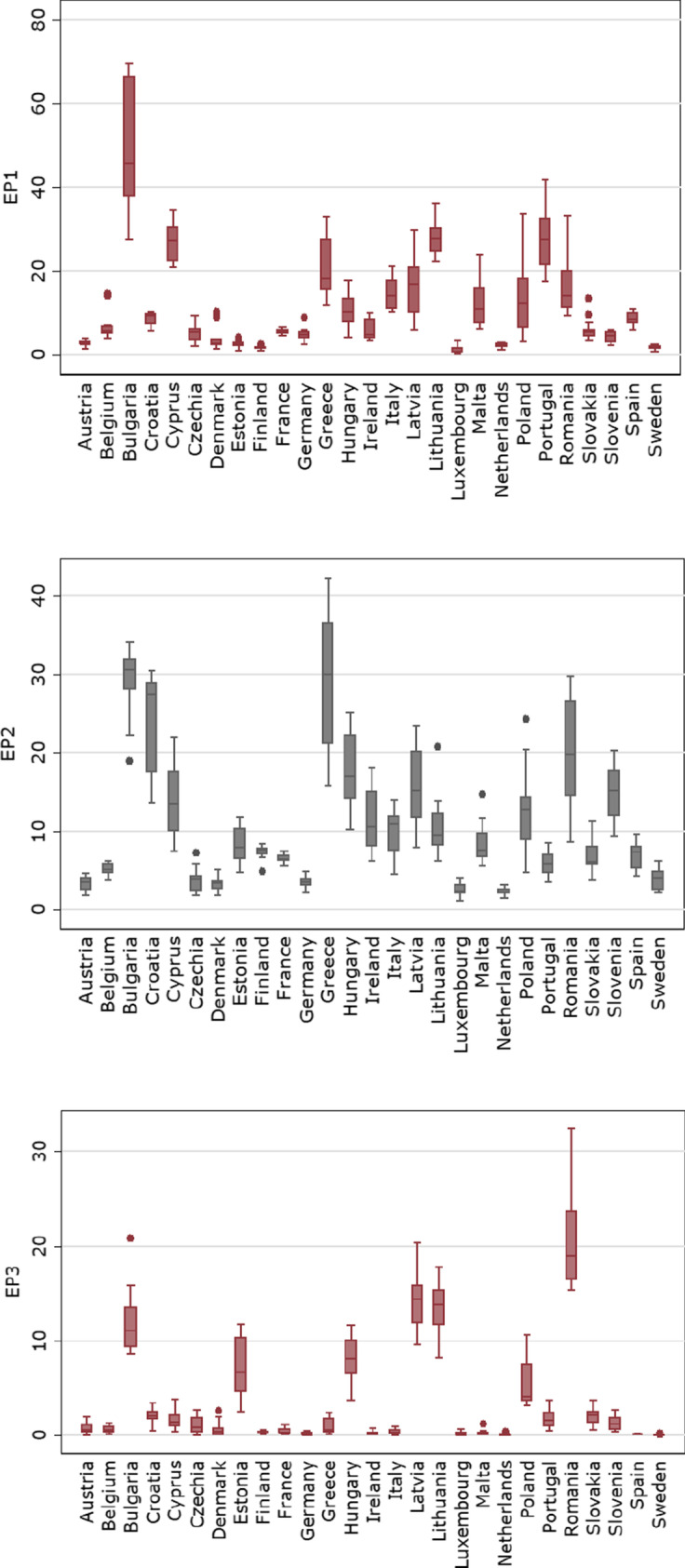
Distribution of energy poverty indicators across EU member states (reference period 2005*–*2020)

To show the distribution and the dispersion of the data for the EP indicators within EU countries, we illustrate the boxplots in Fig. 1. We can then detect how the values of EP indicators are spread out for each country over the reference period. Overall, the distribution of EP indicators is asymmetrical, implying the countries’ deviations over time. Evidently, the distribution of population unable to keep their home adequately warm in Bulgaria is higher relative to the EU members. Even though the affordability of heating services was decently improved in the 2010s, Bulgaria is dependent on the use of natural gas and coal for electricity generation and supply which are imported from Russia. In this view, any disruption on supply will dramatically affect the Bulgarian economy, due to the increase in electricity prices and the unexpected shortages. Verily, the dramatic rise in electricity prices makes the costs for electricity production challenging, which is followed by adverse effects on vulnerable consumers. Conversely, countries such as Austria, Estonia, Netherlands, Luxembourg, Finland and Sweden maintain lower levels of energy poverty, in favour of energy services affordability. The financial conditions of Austrian households evidence the sustained efforts of government to protect poorer households, in the matter of energy services expenditures. Moreover, Luxembourg has set initiatives for the provision of financial aid and support for low-income persons who are unable to meet energy costs, since 2009. An illustration of these programmes is the administration of zero-interest bank loans or the consulting towards a more efficient energy use, and the replacement of energy-guzzling devices with a subsidy of the acquisition cost up to 75%. Although Estonia’s households’ expenditures for heating are high, because of the weather conditions, i.e. the cold climate, the country maintains lower shares of people unable to afford heating services than other EU members. However, the inability to access adequate warming relative to the income conditions in urban areas exceeds that of rural areas. Soever, the relatively low energy costs and the stabilization of electricity prices since 2013 significantly reduce the prospective risks of energy poverty in Estonia. Considering the arrears of utility bills, Greece has experienced severe problems in meeting payment needs for mortgage; rent; utility bills including electricity, natural gas, water and waste; or hire purchase payments. The Greek economic has faced a serious disruption anent economic instability and political turmoil, followed by the Great Recession. As a result, households’ financial ability was in a ruinous state, intensifying the affordability of expenses. Croatia maintains a higher share of the population with difficulties on utility bills relative to the EU average, which evinces a limited purchasing power of Croatian residents. With respect to material deprivation of housing, the distribution of Romania is higher relative to other EU member countries, which implies that an overwhelming number of households suffer from three out four housing dimension items. Recall that the indicator we exploit for material deprivation of housing is based on three of the following items: (a) leaking roof, damp walls/floors/foundation or rot in window frames; (b) accommodation too dark; (c) no bath/shower; and (d) no indoor flushing toilet for sole use of the household. Hence, Romanian private households face severe difficulties in their dwelling. Similarly, a large portion of population in Latvia and Lithuania is considered as materially deprived in the matter of housing. Greece, Bulgaria, Latvia and Lithuania are among the EU members whose citizens are severely material deprived, in terms of dwellings issues. Howbeit, deprivation in Bulgaria has surpassed all the other members, which is followed by the fact that Bulgaria’s social policy measures have failed to reduce inequalities. Meanwhile, the estimation of costs of living is a precondition for the implication of effective policy measures and schemes for material deprivation rates of housing.

## Results and discussion

### Analysis of whole panel convergence

**Fig. 2:**
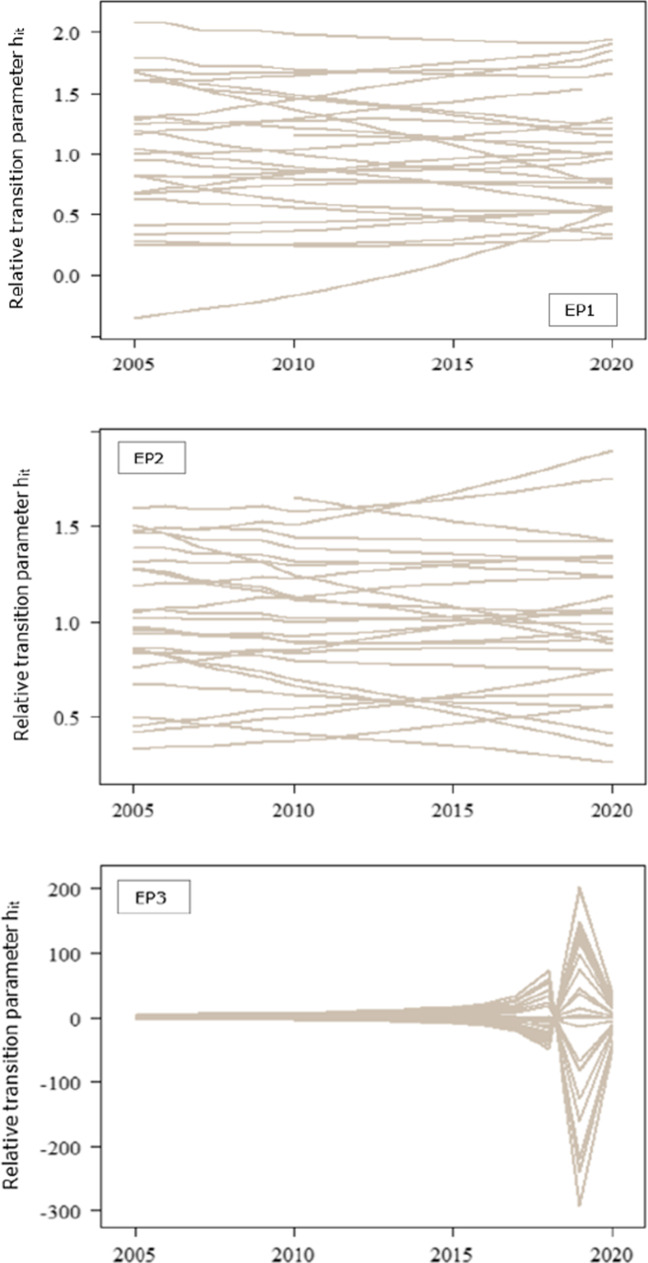
Relative transition paths of energy poverty indicators

Table 2 illustrates the results of the log-*t* regression test for each indicator, respectively. Recall that the log-*t* regression test is a left-tailed *t* test in which the critical value at the 5% level of significance is − 1.65. The null hypothesis of the whole panel convergence (Ho: $\beta \ge 0$) is rejected if the *t*-statistic under the null hypothesis, i.e. ${t}_{\widehat{\beta }}$, is smaller than − 1.65. Hence, if ${t}_{\widehat{\beta }}<-1.65$, we reject the convergence hypothesis at the 5% level of significance and we infer that countries diverge in the long run. The null hypothesis of the whole panel convergence for the first indicator is rejected, that is the *t*-statistic of the estimated regression coefficient ${\widehat{\beta }}_{1}$ is below the − 1.65 critical value, i.e. ${t}_{{\widehat{\beta }}_{1}}=-28.545<-1.65$. Accordingly, the null hypothesis of convergence in the second and third indicators between EU countries is rejected, in that ${t}_{{\widehat{\beta }}_{2}}=-60.306<-1.65$ and ${t}_{{\widehat{\beta }}_{3}}=-4.981<-1.65$. We then infer that the European member states exhibit a divergence behaviour related to the population unable to keep home adequately warm, the arrears on utility bills and the material deprivation for housing. The relative transition paths of energy poverty indicators illustrate the divergence behaviour of EU member states, that is countries move towards different steady states in the long run (Fig. 2). To be more precise, each line corresponds to the relative transition parameter of a given member state for the reference period 2005–2020. The EU economies exhibit a convergence behaviour in specific periods, that is when the relative transition curves cross, which captures the transitional behaviour. In the aggregate, the union shows disparities, withal. Therefore, the European countries have, so far, failed to jointly reach a minimum level of the individuals who are unable to keep their home adequately warm, who are in arrears with utility bills and who are materially deprived in terms of the housing dimension.

**Table 2:** Log-*t* regression test for convergence

Indicator	$\widehat{\beta }$	se $\left(\widehat{\beta }\right)$	${t}_{\widehat{\beta }}$
EP1	0.765	0.027	− 28.545
EP2	− 1.101	0.018	− 60.306
EP3	− 6.753	1.356	− 4.981

EP1, population unable to keep their home adequately warm; EP2, arrears of utility bills; EP3, material deprivation for the housing dimension

### Analysis of the clustering algorithm for convergence clubs

#### Log-t regression test

Nevertheless, the rejection of the whole panel convergence in energy poverty does not entail the inexistence of individual clusters between the states. To detect potential convergence clusters, we conduct the club clustering algorithm, and we summarize the results in Tables 3, 4 and 5. Recall that, after the initial clusters are defined, the algorithm performs multiple iterations for potential merged clusters until the final clusters are detected. The first part of the tables shows the initial clusters, in which the numbers in brackets indicate the number of countries in each cluster. To be more precise, the countries are initially sorted by descending order, and the P&S algorithm conducts multiple log-*t* regression tests to detect the core group. The algorithm adds one country at a time to a core group, until the null hypothesis is not rejected, i.e. member *i* does not converge to the core group. Following this procedure, the log-*t* regression test is conducted by adding each one of the residual members in a new cluster, until the null hypothesis is not rejected. These steps are repeated right up until the initial clusters are extracted.

**Table 3:** Estimation results of the club clustering algorithm (EP1, heating affordability)

Clusters	$\widehat{\beta }({t}_{\widehat{\beta }})$
Initial clubs	
Club 1 [8]	0.124 (1.451)
Club 2 [10]	− 0.040 (− 0.501)
Club 3 [9]	0.203 (1.688)
Club merging	
Club 1 + 2	− 0.392 (− 8.618)
Club 2 + 3	− 0.573 (− 16.943)
Final clubs	
Club 1 [8]	0.124 (1.451)
Club 2 [10]	− 0.040 (− 0.501)
Club 3 [9]	0.203 (1.688)

The numbers in brackets indicate the number of countries in the clubs. The initial classification suggests 3 clubs, while the iterations come in 3 convergence clubs

**Table 4:** Estimation results of the club clustering algorithm (EP2, arrears in utility bills)

Clusters	$\widehat{\beta }({t}_{\widehat{\beta }})$
Initial clubs	
Club 1 [5]	0.001 (− 0.021)
Club 2 [6]	0.084 (− 0.833)
Club 3 [9]	0.179 (− 2.254)
Club 4 [2]	0.532 (− 18.325)
Club 5 [2]	− 3.004 (− 1.139)
Club 6 [2]	0.142 (− 1.153)
Club 7 [1]	–
Club merging	
Club 1 + 2	− 0.129 (− 2.465)
Club 2 + 3	− 0.263 (− 5.042)
Club 3 + 4	0.037 (− 0.64)
Club 4 + 5	− 1.697 (− 7.740)
Club 5 + 6	− 1.503 (− 9.973)
Club 6 + 7	− 1.902 (− 60.220)
Final clubs	
Club 1 [5]	0.001 (− 0.021)
Club 2 [6]	0.084 (− 0.833)
Club 3[11]	0.037 (− 0.64)
Club 4[2]	− 3.004 (− 1.139)
Club 5[2]	0.142 (− 1.153)

The numbers in brackets indicate the number of countries in each club. The initial classification suggests 6 convergence groups and one divergent group, while the iterations yield 5 final convergence clubs

**Table 5:** Estimation results of the club clustering algorithm (EP3, material deprivation)

Clusters	$\widehat{\beta }({t}_{\widehat{\beta }})$
Initial clubs	
Club 1 [5]	1.899 (− 30.945)
Club 2 [7]	0.152 (− 1.309)
Club 3 [15]	4.632 (− 2.566)
Club merging	
Club 1 + 2	0.745 (− 7.918)
Club 2 + 3	− 2.82 (− 1.902)
Final clubs	
Club 1 [12]	0.745 (− 7.918)
Club 2 [15]	4.632 (− 2.566)

The numbers in brackets indicate the number of countries in each convergence club. The initial classification yields 3 groups, while the iterations result in 2 final convergence clubs

The initial classification of EP1 suggests three clusters (Table 3). Precisely, the estimated $\widehat{{\beta }_{1}}$ coefficients of the log-*t* regression tests for the initial clusters 1, 2 and 3 are statistically insignificant, in that the estimated *t*-statistics are greater than the 5% critical value, i.e. ${t}_{\widehat{{\beta }_{1}}}=1.451>-1.65$, ${t}_{\widehat{{\beta }_{1}}}=-0.501>-1.65$ and ${t}_{\widehat{{\beta }_{1}}}=1.688>-1.65$. This implies that the null hypothesis of convergence for the initial clusters is not rejected; hence, 8, 10 and 9 EU member states define the first, the second and the third initial clusters, respectively. Given the identification of the initial clusters, the P&S algorithm investigates whether the initial clusters can be merged into a larger cluster. For each pair of the initial clubs, the test of club merging, i.e. the log-*t* regression test, is iterated until no more clubs can be merged into a larger convergence group. Precisely, each member of the initial club 2 is added in the initial club 1 and the log-*t* regression test is anew conducted. The test of club merging between the initial clubs 1 and 2 yields ${t}_{\widehat{\beta }}=-8.618<-1.65$, implying the rejection of the null hypothesis, i.e. clubs 1 and 2 are not merged into a larger group. The test of club merging is repeated for the initial clubs 2 and 3, successively. The log-*t* regression test gives ${t}_{\widehat{{\beta }_{1}}}=-16.943<-1.65$, and the null hypothesis is rejected, i.e. clubs 2 and 3 diverge. Finally, the initial clusters define the final groups as the club merging is not feasible. Hence, the classification suggests that three groups of countries converge to an equilibrium steady state of the population unable to keep their home adequately warm.

Table 4 shows the results of the arrears in utility bills. Against the small number of initial clubs of the affordability for heating services, the initial classification of the arrears in utility bills (EP2) evinces six convergence clubs and one divergence club. Accordingly, we observe that the initial clusters 1, 2, 3, 4, 5 and 6 comprise 5, 6, 9, 2, 2 and 2 members, respectively. The estimated $\widehat{{\beta }_{2}}$ coefficients of the preliminary log-*t* regression tests are statistically insignificant, that is given the non-rejection of the null hypothesis, the regression parameters are equal or greater than zero. To that end, the algorithm provides strong evidence for the existence of six initial convergence groups of countries and one divergent member. Analytically, the estimated *t*-statistics for the initial clubs 1–6 are greater than the 5% critical value, i.e. ${t}_{\widehat{{\beta }_{2}}}=0.021>-1.65$, ${t}_{\widehat{{\beta }_{2}}}=0.833>-1.65$, ${t}_{\widehat{{\beta }_{2}}}=2.254>-1.65$, ${t}_{\widehat{{\beta }_{2}}}=18.325>-1.65$, ${t}_{\widehat{{\beta }_{2}}}=-1.139>-1.65$ and ${t}_{\widehat{{\beta }_{2}}}=1.153>-1.65$. This indicates that the null hypothesis of convergence for the initial clusters is not rejected, and 5, 6, 9, 2, 2 and 2 EU member states form the first, the second, the third, the fourth, the fifth and the sixth initial clusters, individually. In turn, the algorithm performs a step-by-step club merging procedure. The test of club merging between the initial clubs 1 and 2 gives ${t}_{\widehat{{\beta }_{2}}}=-2.465<-1.65$, which implies the rejection of the null hypothesis; viz., the initial clubs 1 and 2 diverge. As a result, we extract the final convergence club 1, which corresponds to the initial club 1. Subsequently, the test for clubs 2 and 3 suggests the rejection of convergence hypothesis, being that ${t}_{\widehat{{\beta }_{2}}}=-5.042<-1.65$; hence, we have the second final convergence club, i.e. club 2, which originates from the initial club 2. Identically, the algorithm performs the test for the initial clubs 3 and 4 and the results cannot reject the null hypothesis of convergence, as ${t}_{\widehat{{\beta }_{2}}}=0.640>-1.65$. Consequently, clubs 3 and 4 are merged into a larger group with [9] + [2] = 11 members, which form the third final convergence club. In this step, the algorithm is at a critical juncture, as clubs 3 and 4 are merged. Nevertheless, the algorithm follows the steps for the initial clubs 4 and 5, giving an estimate of ${t}_{\widehat{{\beta }_{2}}}=-7.740<-1.65$. Accordingly, the clubs diverge and we get the final convergence club 4, which corresponds to the initial club 5. At the last step, the log-*t* regression test is performed for the initial clubs 5 and 6, which finally suggests the rejection of convergence, that is ${t}_{\widehat{{\beta }_{2}}}=-9.973<-1.65$. Alternatively, the clubs diverge and the algorithm estimates the final convergence club 5, i.e. the initial club 6. Ultimately, we find five convergence clubs of countries in the arrears in utility bills. Overall, the initial clusters 3 and 4 are merged into a larger group, while the remaining initial clusters fail to merge. Hence, the residual initial clusters define the final clusters, individually. The algorithm finally estimates five groups of countries that converge and one country that diverges from the others in the arrears on utility bills.

Table 5 illustrates the results of material deprivation for the housing dimension. Three convergence groups of countries are initially formed, including 5, 7 and 15 members, respectively. The log-*t* regression test suggests the existence of three initial clusters since the estimated $\widehat{{\beta }_{3}}$ coefficients are statistically insignificant, i.e. the null hypothesis in favour of convergence is not rejected. Precisely, the estimated *t*-statistics for the initial clubs 1–3 are greater than the 5% critical value, that is ${t}_{\widehat{{\beta }_{3}}}=30.945>-1.65$, ${t}_{\widehat{{\beta }_{3}}}=1.309>-1.65$ and ${t}_{\widehat{{\beta }_{3}}}=2.566>-1.65$. Then, the club merging algorithm is conducted to detect whether the initial clubs merge into larger groups. The log-*t* regression test between the initial clubs 1 and 2 cannot reject the null hypothesis of convergence because ${t}_{\widehat{{\beta }_{3}}}=7.918>-1.65$. Hence, the initial clusters 1 and 2 merge into another club and define the final convergence club 1 with [5] + [ 7] = 12 countries. Subsequently, the test between the initial clubs 2 and 3 suggests the rejection of convergence, given that ${t}_{\widehat{{\beta }_{3}}}=-1.902<-1.65$. Hence, we find the second final convergence club which corresponds to the initial club 3. Finally, the union exhibits two groups of countries that converge to an equilibrium steady state in material deprivation for the housing dimension.

#### Identification of members

In this section, we analyse the empirical results, and we seek to interpret the patterns of convergence between the members. We should originally identify which members compose the clusters, and the distribution of energy poverty indicators between them. Table 6 delineates the EU member states in each group, as well as the level of energy poverty. Considering EP1, we find that the “High” EP1 club consists of Bulgaria, Cyprus, Greece, Ireland, Italy, Lithuania, Luxembourg and Portugal and the “Moderate” EP1 club consists of Croatia, France, Germany, Hungary, Latvia, Malta, Netherlands, Romania, Slovakia and Spain. Verily, the EU-wide survey of 2018 for the population unable to keep their home adequately warm shows that Bulgaria, Lithuania, Greece, Cyprus, Portugal and Italy lie around the upper threshold of the distribution. Moreover, the final convergence club of EP1, i.e. the “Low” EP1 cluster, incorporates the following states: Austria, Belgium, Czechia, Denmark, Estonia, Finland, Poland, Slovenia and Sweden. The EU-wide survey of 2018 argues that Austria, Finland, Estonia and Sweden are among the countries whose individuals manifested the lowest inability to afford heating service.

**Table 6:** Convergence clubs of energy poverty indicators

	Countries
Population unable to keep home adequately warm	
High	Bulgaria, Cyprus, Greece, Ireland, Italy, Lithuania, Luxembourg, Portugal
Moderate	Croatia, France, Germany, Hungary, Latvia, Malta, Netherlands, Romania, Slovakia, Spain
Low	Austria, Belgium, Czechia, Denmark, Estonia, Finland, Poland, Slovenia, Sweden
Arrears on utility bills	
High	Bulgaria, Croatia, Cyprus, Romania, Spain
Above average	Denmark, Finland, Hungary, Ireland, Latvia, Slovenia
Average	Austria, Belgium, Estonia, France, Italy, Lithuania, Luxembourg, Malta, Poland, Portugal, Slovakia
Below average	Germany, Sweden
Low	Czechia, Netherlands
Divergent group	Greece
Material deprivation rate of housing	
High	Croatia, Estonia, Germany, Hungary, Ireland, Latvia, Lithuania, Luxembourg, Malta, Poland, Slovakia, Sweden
Low	Austria, Belgium, Bulgaria, Cyprus, Czechia, Denmark, Finland, France, Greece, Italy, Netherlands, Portugal, Romania, Slovenia, Spain

Against the number of convergence clubs for EP1, EP2 showcases 5 convergence groups and one divergence group. Given the number of clusters, we set a 5-point Likert scale for the volume of arrears in utility bills, viz., “Very high”, “Above average”, “Average”, “Below average” and “Very low”. Accordingly, we estimate that the very high EP2 group consists of Bulgaria, Croatia, Cyprus, Romania and Spain, while the above average cluster consists of Denmark, Finland, Hungary, Ireland, Latvia and Slovenia. However, we should clarify that utility bills apply not only to energy; rather, energy bills are the principal component of invoices paid for essential services. We observe that Bulgarian households suffer from difficulties on utility payments and these members are unable to keep energy costs down. Despite, Austria, Belgium, Estonia, France, Italy, Lithuania, Luxembourg, Malta, Poland, Portugal and Slovakia constitute the average EP2 club, that is the countries maintain moderate levels of difficulties on utility bills. Moreover, Germany and Sweden define the below average EP2 club and Czechia and Netherlands lie around the lower threshold of the distribution, i.e. very low EP2.

Instead, the results of material deprivation rate for housing are narrow, that is the EU countries formulate two convergence clubs. High EP3 club includes Croatia, Estonia, Germany, Hungary, Ireland, Latvia, Lithuania, Luxembourg, Malta, Poland, Slovakia and Sweden, rather Austria, Belgium, Bulgaria, Cyprus, Czechia, Denmark, Finland, France, Greece, Italy, Netherlands, Portugal, Romania, Slovenia and Spain join the low EP3 club. Evidently, any geography-specific patterns across energy poverty convergence clubs are hard to determine. However, we provide potential explanations in the ensuing section.

**Fig. 3:**
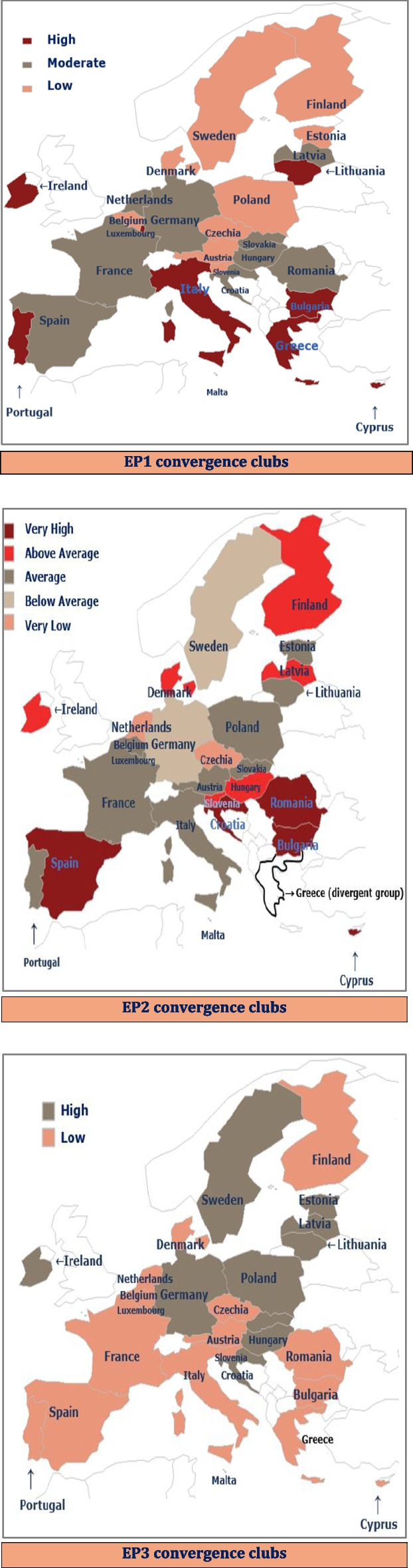
Spatial distribution of energy poverty convergence clubs

Figure 3 illustrates the spatial distribution of energy poverty convergence clubs between the EU member states. The distribution of population unable to keep home adequately warm is uniform in the European Union. In other terms, the number of countries in each cluster is approximately equal. Indeed, the population in 8, 10 and 9 out of 27 EU members indicates high, moderate and low inability to afford heating services. Hence, we infer that the share of individuals that suffer from the inability to maintain their home warm is uniformly dispersed across countries. Nevertheless, this does not apply in reference to the arrears on utility bills. Evidently, we estimate that 11 out of 27 EU countries show average difficulties on utility payments, but [5] + [6] = [11] out of 27 members indicate “very high” and “high” arrears, respectively. Concisely, we need to highlight that most of the European households face severe and moderate difficulties on utility bills. Considering the material deprivation for housing, we find that the distribution is uniform between EU countries; viz., 12 out of 27 and 15 out of 27 members indicate high and low deprivation. In view of this, the persons who mostly or slightly live under the risk of material deprivation for housing are equally distributed between the European economies.

#### Descriptive statistics

We present the descriptive statistics of energy poverty indicators between convergence clubs in Table 7. The descriptives are extracted from the original data, i.e. exclusive of any standardization or detrending processes. With respect to EP1 indicator, we observe that the average energy poverty reduces with the clubs, viz., ${\overline{\mathrm{EP}1} }_{\mathrm{c}1}>{\overline{\mathrm{EP}1} }_{\mathrm{c}2}>{\overline{\mathrm{EP}1} }_{\mathrm{c}3}$. Nevertheless, due to the asymmetry in the original data of EP1, the sample mean is not representative for the inference. To that end, we should account for the median and the third quartile which are kindly consistent with the skewness. If we consider for the second (median) and third (Q3) quartiles, we get 22.1% and 29.3%, respectively. In other words, the values of population unable to keep their home adequately warm are below 22.1% across 50% of the observations (60 out of 120) and below 29.3% across 75% of the observations (90 out of 120) in the first club. We observe that the median and the third quartile decrease as we move from club 1 to 3. Verily, the values of the inability to afford heating services are below 0.3% across 50% of the observations (80 out of 160) and below 5.6% across 75% of the observations (120 out of 160) in the third club. This explains our reasoning behind the definition of the 3-point Likert scale from High to Low, as well as of EP2 and EP3 Likert scale points. Given the descriptives of arrears in utility bills, we find that the values of the indicator are below 17.5% across 50% of the observations (40 out of 80) and below 28% across 75% of the observations (60 out of 80) in the first convergence club, i.e. in the very high EP2 club. Instead, 50% of the values within the moderate EP2 club are below 6.3% and 75% of them are below 9.4%. Conversely, the members to which households are relieved from arrears on utility bills showcase lower values of quartiles related to the other countries. Concerning material deprivation for housing, we detect two convergence clubs, i.e. high and low EP3 clusters. At any rate, we find that 96 out of 192 observations in the high material deprivation club receive values below 2.2%, while this percentage is 0.6% for the low EP3 cluster. From a broad perspective, the quartiles indicate that material deprivation for the housing dimension in the first club is higher related to the second club.

**Table 7:** Descriptive statistics of energy poverty indicators across convergence clubs

	Countries	*N*	Mean	SD	Min	Max	Median	Q3
EP1, population to keep home adequately warm (%)								
Club 1	[8]	120	22.1	15.8	0.3	69.5	22.1	29.3
Club 2	[10]	160	9.1	5.9	1.3	33.3	7.5	10.9
Club 3	[9]	144	4.8	4.9	0.9	33.6	0.3	5.6
EP2, arrears on utility bills (%)								
Club 1	[5]	80	18.5	9.3	4.3	34	17.5	28
Club 2	[6]	96	11.7	6.2	1.8	25	11.2	17.1
Club 3	[11]	176	7.2	3.8	1.1	24.2	6.3	9.4
Club 4	[2]	32	3.7	1	2.2	6.2	3.6	4.5
Club 5	[5]	32	3	1.3	1.5	7.2	2.5	3.9
EP3, material deprivation for housing (%)								
Club 1	[12]	120	22.1	15.8	0.3	69.5	22.1	29.3
Club 2	[15]	160	9.1	5.9	1.3	33.3	7.5	10.9

### Interpretation of relative transition paths

#### Population unable to keep home adequately warm (EP1)

**Fig. 4:**
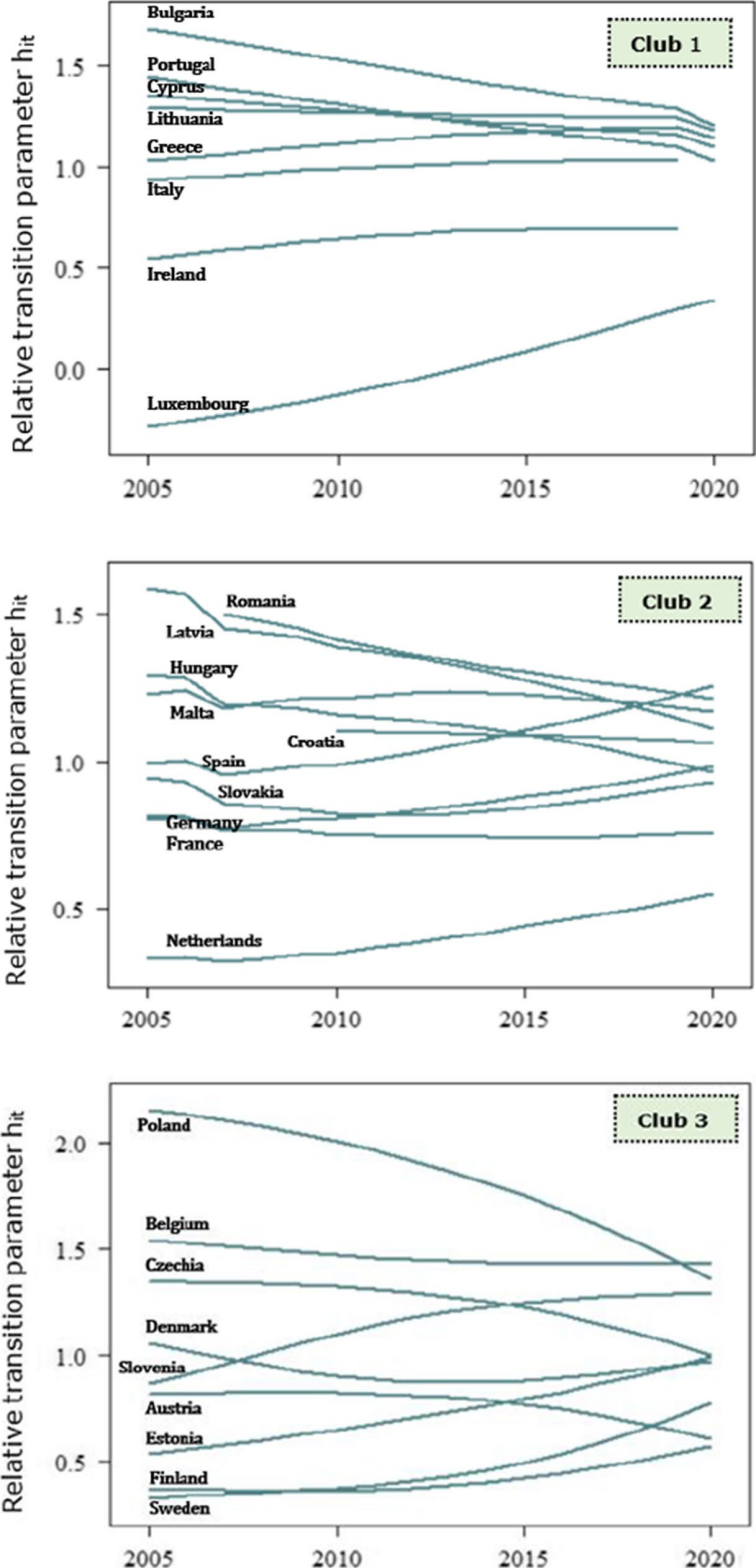
Relative transition paths of the EP1 indicator across convergence clubs

**Fig. 5:**
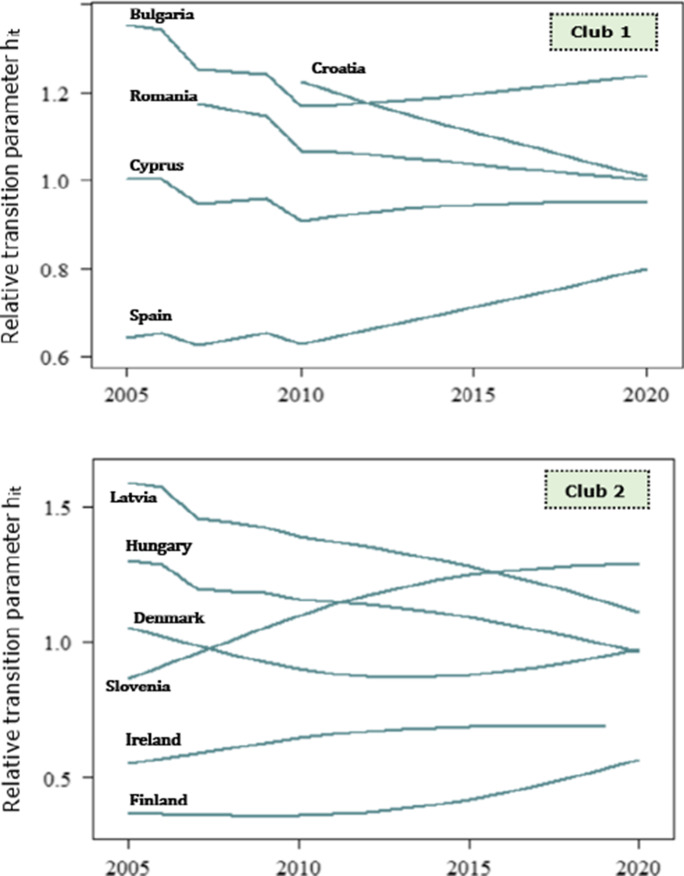

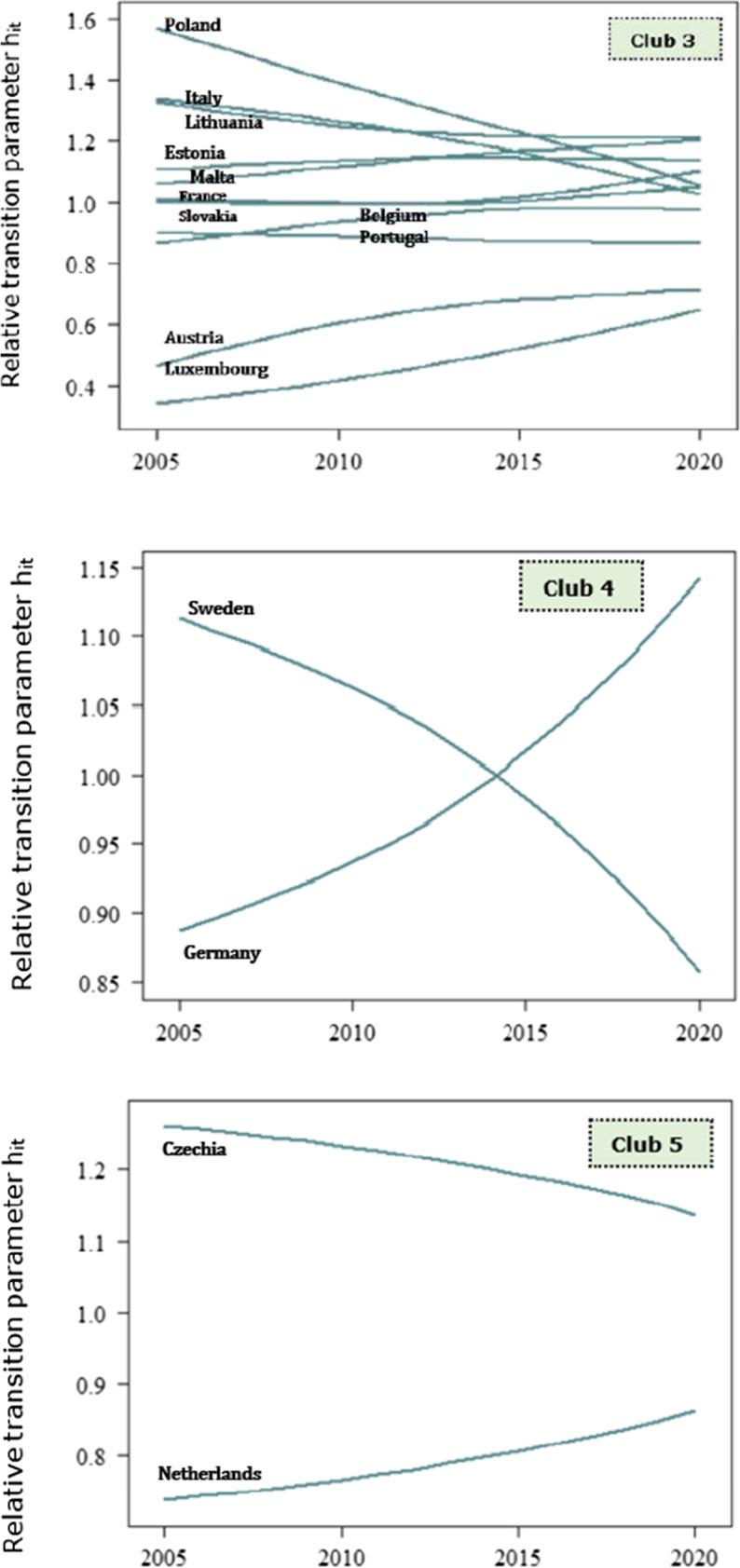
Relative transition paths of the EP2 indicator across convergence clubs

**Fig. 6:**
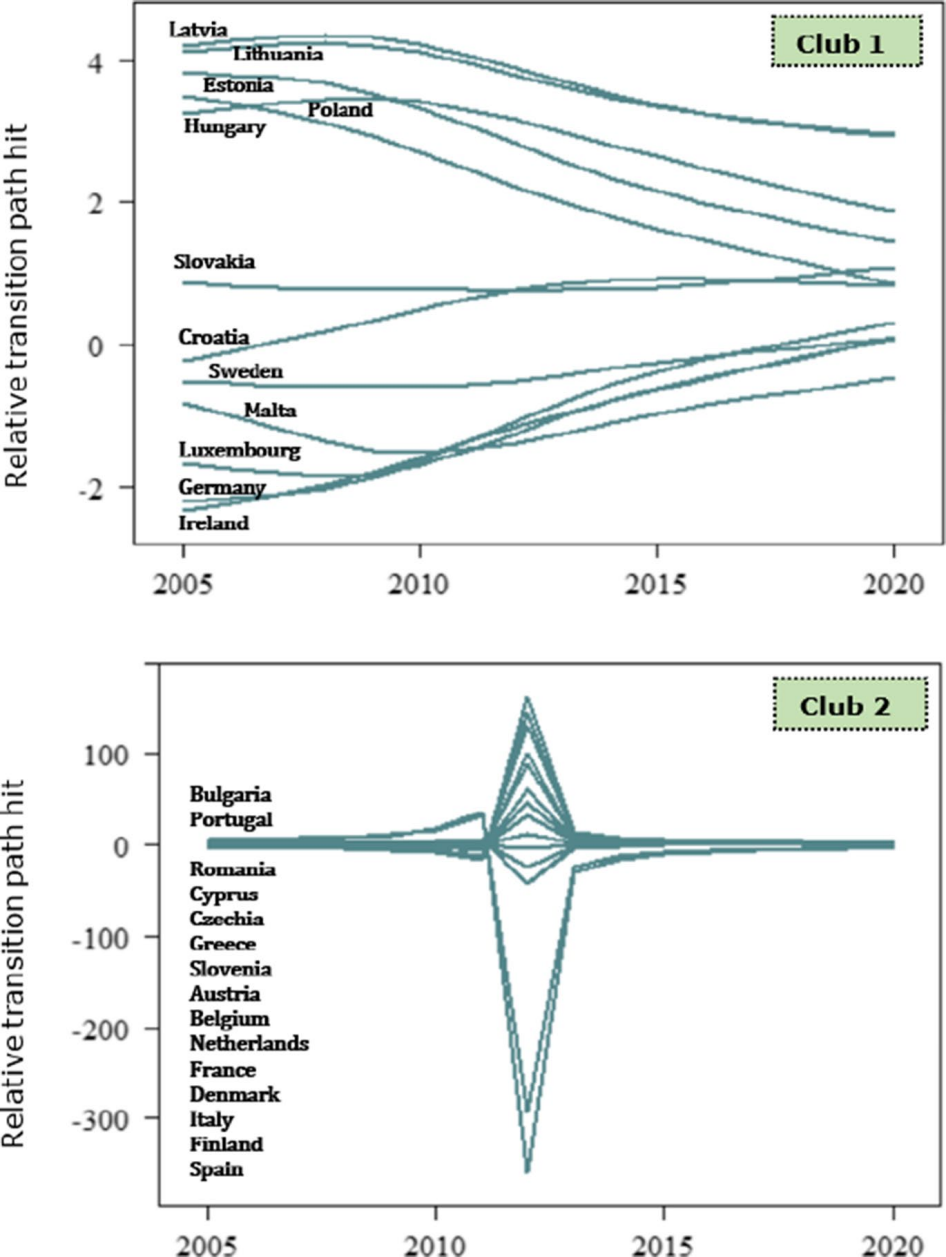
Relative transition paths of the EP3 indicator across convergence clubs

The transitional behaviour of countries is accurately illustrated by the relative transition paths across clubs in Figs. 4, 5 and 6. The plot of relative transition paths is a time series plot of the relative transition parameter *h*_*it*_, for country *i* at time *t*. Recall that *h*_*it*_ captures the transitional behaviour of country *i* with respect to the cross-sectional average at time *t*. As *h*_*it*_ limits to unity, the economies converge to an equilibrium steady state; instead, the economies showcase a divergence behaviour if *h*_*it*_ limits to zero. From a broad perspective, we observe that the relative transitional parameters between EU members in the same club converge to an equilibrium steady state in the long run.

At first, we analyse the transitional behaviour of EU members for the population unable to keep their home adequately warm (see Fig. 1). The relative transition paths of Cyprus, Portugal and Lithuania cross over in 2010, and the countries indicate an even speed of convergence until 2013. This implies that the economies converge to a different steady state relative to the other countries in the high EP1 group (i.e. club 1) between the reference period. Instead, Cyprus and Portugal follow the same paths until 2016, while Portugal moves towards a different path. Soever, the countries converge to a steady state in the long run.

Particularly, Bulgarian households are mostly hit by energy poverty, in the matter of heating services affordability, and the economy is among the members with the lower speed of convergence related to other members in the first cluster. Individuals who reside in countries with intense climate conditions are significantly affected by temperature outcomes (Beg et al., 2002; Santamouris & Kolokotsa, 2015). Verily, the quality of life and the living standards of the vulnerable consumers are adversely affected by extreme weather conditions (Thomson et al., 2019).

Precisely, the affordability of energy needs for the Bulgarian households is inherently affected by temperature, that is, the country showcases extremely high and low temperatures during summer and winter, respectively (Hajdinjak & Asenova, 2019). As a result, individuals should spend most of their income on winter, to keep home warm, and on summer, to access cooling services. Although Portugal indicates moderate temperatures, several households suffer from the inability to maintain heating services, while the country is one of the most vulnerable economies in the European Union (Bollino & Botti, 2017; Bouzarovski & Tirado Herrero, 2017). However, this can be attributed to significant insulation issues, because of the age and the low efficiency of the residential building stock. Precisely, most of the Portuguese buildings are old and the residential buildings can hardly conserve thermal energy due to poor thermal characteristics and low efficiency (Mafalda Matos et al., 2022).

Moreover, Greece, Romania, Portugal and Italy are mostly hit hard by the general upward trend on electricity prices, which is followed by network charges and taxes. Instead, to provide a potential explanation on the formation of high EP1 club, we should account for the housing conditions. Several European households are suffering from severe structural problems, which, in turn, affect the ability to keep their home adequately warm (Dubois & Meier, 2016). Based on the EU-SILC, Portugal, Italy, Lithuania and Greece are among the EU members with the highest rates of severe housing deprivation, i.e. a significant share of population suffers from leaking roof or darkness in the dwelling, or a lack of bath or toilet. The inefficiencies in dwellings and the population growth have significantly deteriorated persons’ inability to afford energy services and housing costs. This is evident for the citizens in Luxembourg, where structural problems on dwellings are intense across households, and particularly between the youth. Moreover, the severe housing conditions are reflected in the great number of decayed buildings and the poor thermal characteristics (Karpinska & Śmiech, 2020).

Conversely, the number of the intersecting points of relative transition curves in the moderate EP1 club (club 2) exceeds the respective number of high EP1 club. Specifically, this implies that countries diverge or converge to different steady states at specific periods. In the early stages of energy poverty, that is between 2005 and 2008, France and Germany converge to a steady state, while the relative transition paths diverge thereon. However, Latvia and Lithuania follow the same path in the main. Even though the relative transition parameters fluctuate over the reference period, they evidently move towards the equilibrium steady state on the long run. This is certainly true in the case of Malta, to which the relative transition parameter decreases in 2007, increases between 2008 and 2012 and then follows a downward trend, i.e. the economy moves towards the panel average. Instead, Slovakia diverges from the panel average until 2012 and EP1 finally returns to the panel mean. A certain share of households in Romania, Latvia and Hungary is also suffering from problems in dwellings, but the results show that these countries maintain moderate inability to keep home adequately warm. Alternatively, the economies in the moderate EP1 club indicate a mixed performance on the ability to afford heating services.

However, Hungary maintains the lowest energy prices in the EU which contributes to the mitigation of the adverse effects on vulnerable consumers, i.e. particularly single-parent families, unemployed or single-elderly persons and large families. Besides, the issues on housing deprivation are significantly attributed to the inefficiencies in the country’s energy supply system (Bajomi et al., 2020). Based on the EU-SILC surveys, economies in the moderate EP1 club showcase lower rates of severe housing conditions, rather than countries in the first club. Nevertheless, a large part of the energy-poor in France, Germany, Croatia, Spain and Malta is attributed to social housing and private tenants’ tenures. In essence, disaggregated data for these countries suggest that people who live in apartment-type dwellings are considered more vulnerable, related to those who reside in semi-detached or detached dwellings. Besides, this can be partly explained by the severe effects of financial distress, the outbreak of the global health crisis and the poor energy efficiency of buildings.

Against the fact that the relative transition paths of the moderate EP1 club osculate to each other at specific periods, the paths of low EP1 club intersect per two countries. For instance, we find that Poland and Belgium, Czechia and Slovenia, Denmark and Slovenia, Austria and Estonia, as well as Austria and Finland indicate a crossover point at specific years. Overall, the third club of EP1 includes countries that prosper in terms of the access to heating services. Economies such as Austria, Belgium, Sweden and Finland indicate higher performance than the EU average, in terms of energy poverty. Particularly, just a small portion of the Finish and Swedish population reports that they are unable to keep their home sufficiently warm. This can be attributed to the lease agreements, i.e. the fact that rental payments are frequently inclusive of energy bills, implying that the median share of the households’ energy costs on total income lowers. Nevertheless, this does not necessarily entails that energy poverty reduces; instead, energy expenditures of households are not deeply affected by rising energy prices. Moreover, Belgium, Sweden, Finland and Estonia set disconnection protection schemes during heating and winter periods. As a result, the population unable to keep home adequately warm significantly reduces.

We then infer that the inability to keep home adequately warm across convergence clubs is potentially attributed to structural conditions of housing, weather conditions (i.e. temperature outcomes) and energy costs. Ultimately, economies of which households are hit hard by the inability to keep home adequately warm, i.e. countries in the high energy poverty club, are severely affected by soaring energy prices and partially affected by weather conditions. Individuals who reside in countries that signal moderate energy poverty are also distressed by energy prices, but to a lesser extent withal. Howbeit, households in these economies showcase severe problems on buildings stock, i.e. the households suffer from structural problems which intensify the inability to access heating services. In contrast, economies that prosper in terms of the ability to afford heating services set disconnection protection schemes, that is support income and social schemes, in favour of the access to energy services. Besides, the median share of energy expenditures in households’ income is below the EU median, which is mainly attributed to the inclusion of energy bills on rental payments.

#### Arrears on utility bills (EP2)

Against the lower number of convergence clubs for the inability to keep home adequately warm, EU countries form a greater number of clusters in terms of the arrears on utility bills (Fig. 2). Foremost, we observe that the relative transition paths of the EU members within the third club, i.e. middle arrears on utility bills, indicate the highest number of intersecting points related to the other clubs. To be more precise, this implies that even though countries in the middle club converge to different steady states at specific periods, they converge to an equilibrium state in the long run. Nevertheless, just 4 out of 27 EU members form the low and very low convergence clubs, which signifies that a large part of the European households struggle with arrears on utility bills. Given the relative transition paths of the very high EP2 club (i.e. club 1), Bulgaria and Croatia converge to a steady state in 2012, but follow different paths thereon. At the early stages of transitional behaviour, that is in 2010, the relative parameters fluctuate; instead, the transitional parameters indicate a deterministic trend beyond that year. Howbeit, Bulgaria showcases the lowest speed of convergence with respect to the other members in the club. The financial crisis caused severe effects on employment and disposable income, and therefore, it triggered the arrears on utility bills across Croatian, Romanian, Spanish, Bulgarian and Cypriot households. In tandem, the arrears on utility bills in Romania are significantly affected by climate conditions, that is the country experiences cold climates which raise the energy consumption for heating services. Hence, the energy bills prove less affordable for households. Evidently, we observe that Bulgaria and Cyprus are included in the first convergence club of both EP1 and EP2 indicators. Alternatively, we suggest that these economies are worst-performing among the EU member states, in terms of energy poverty. Instead, the countries in the high EP2 club indicate a greater speed of convergence on average, rather than members in the first club. The adverse effects of financial crisis deteriorated the living and income conditions of Irish and Slovenian households, as a large part of population is at a higher risk of energy poverty. In contrast, economies which form the average EP2 club maintain social policies that mitigate the difficulties on utility bills. France and Italy are substantially setting similar policies that notably reduce the arrears on utility bills. Besides, progressive policies such as social tariffs for energy consumption are considerable in the Belgian economy. Despite, Germany, Sweden, Czechia and Netherlands display the least arrears on utility bills; viz., the economies perform better than the EU average. The notably low levels of difficulties on utility bills for the Swedish and German population are potentially attributed to the policy measures, in favour of energy efficient buildings.

#### Material deprivation rate on housing (EP3)

Interestingly, the relative transition parameters for the material deprivation on housing dimension formulate two convergence clubs, i.e. low and high EP3 clubs. The relative transition paths across countries smoothly converge to the equilibrium steady state. Howbeit, the relative parameters in the second club indicate extreme values for 2019. Verily, at the early stages of transitional behaviour, the curves are being osculated, i.e. the economies showcase a convergence behaviour, but they significantly diverge in 2019, while moving towards the equilibrium state in 2020. The extreme values of the relative transition parameters originate from the large values of France, Belgium, Czechia, Finland, Spain, Austria, Netherlands, Greece and Italy, as well as from the least negative values of Romania, Latvia, Lithuania and Bulgaria. The occurrence of the outliers implies that a large part of the European population suffers severely from structural problems in their homes. Several households across the union are found to lack basic sanitary facilities or struggle with problems such as leaking roofs and damp walls. Overall, households undergo profound issues on the efficiency of basic sanitary facilities and other problems in infrastructural terms.

## Discussion

The share of cost of heating services in the EU households’ final expenditures is high, and as a result, it threatens vulnerable consumers. Consumer vulnerability, energy poverty and the disconnections are among the major challenges of the European members. Recently, energy is an essential source for heating and cooling services, regarding the diverse climates in the European Union. If energy resources in households become unaffordable, the social and economic impacts are severe. Latest arguments of international energy agents and the European Commission consider low income, low energy efficiency on households/dwellings and high energy prices to be the fundamental causes of energy poverty. In view of these considerations, we summarize three broader factors, i.e. the economic, the social and the political perspective of energy poverty.

The effect of institutions and the regulatory framework is exemplified in the Third Energy Package of the European Commission that enters into force in 2009. Verily, the package has been criticised about the regulatory solutions in favour of the private investments in new technologies on the electricity sector. Concurrently, the increasing competition, the uncertainties and the various regulatory failures of the electricity markets affect price signals, i.e. energy prices or the short-term markets.^1^ To that end, the households’ affordability for heating services is significantly affected by uniform policies or country-specific policies. This implies that, instead of the existence of uniform energy policies, the responses of governments in the electricity market’s challenges vary across the European members. The economic state, the policy framework and the socio-political systems have a considerable impact on households’ income, through austerity measures, consumer-related income schemes and energy price regulations. Moreover, the regressive effects of these challenges on vulnerable consumers are strictly associated with the effectiveness of the initiatives and the compensation mechanisms that are exerted by each state. Besides, the rate and the degree by which a European economy comforts into the European regulation system are different across EU countries.

In contrast, a current debate on energy poverty and vulnerability revolves around the definitions of the terms. Verily, in the amendments of 22 June 2022, the European Parliament argued that no standard EU-level definition of energy poverty is existent. As a result, some policy measures and initiatives on energy poverty may prove ineffective in alleviating energy poverty. Besides, the inexistence of a uniform definition impedes the collection of transparent and comparable data. In other terms, an inconclusive dispute over which part of the population is considered vulnerable adversely affects the outcomes of the undertaken initiatives and the level of support on certainly poor households. Energy inequalities are indirectly associated with the provision of an exact and clear definition of energy poverty. In view of this, the core issue is to identify who really needs to receive support, in terms of the access to clear and affordable energy services, similar income support schemes or other aspects of energy poverty. Despite the vagueness of the definition, the European Parliament states that policy measures, such as social tariffs or income support schemes alleviate poverty outcomes in favour of the energy-poor households, but in the short run. Nevertheless, while these measures are effective on short-term poverty, they are unable to support governments’ schemes to fight against poverty in the long run. In terms of the government perspective, the provision of insufficient, or even the lack of sufficient information to consumers about energy use and conservation may also cause adverse effects (Halkos & Gkampoura, 2021a, 2021b). Economies with liberalised markets set initiatives and campaigns to build consumers’ awareness of energy issues. These issues include the efficient exploitation of energy services and products as well as the comparison of energy prices. Hence, consumers’ ignorance of energy-related issues slows down the prosecution of regulatory measures and strategies (Nussbaumer et al., 2012).

The underlying argument in favour of energy poverty alleviation is the adoption of structural measures, i.e. initiatives related to building renovation in terms of comfortable and energy-efficient households and dwellings. Household characteristics such as size, year of residential construction, location and building quality directly affect energy poverty outcomes. The quality and basic amenities such as household equipment, the number of rooms and the existence of energy-efficient appliances affect the energy use (Thomson & Snell, 2013) and, accordingly, the energy bills and the share of energy payments on housing costs. In many cases, a higher number of rooms impede the household’s ability to keep home adequately warm, particularly for low-income households (Thomson & Snell, 2013). Meanwhile, energy poverty outcomes differ between rural and urban areas, i.e. the location may inherently affect the share of energy-poor to the total population. The needs of rural citizens are more income oriented rather than energy oriented, i.e. urbanization increases the energy dependence as the economies focus on economic growth, while residents in rural areas may not be prioritised in spending their disposable income on unaffordable energy services. Moreover, individuals who reside in inefficient and very old homes or use older equipment indicate higher energy consumption rather than people who own energy-efficient appliances and equipment. The problems with infrastructure such as dwellings not comfortably cool during summer or warm during winter, leaking roofs, damp walls/floors/foundations, dark accommodation, problems with indoor flushing toilet for sole use and lack of heating or cooling equipment severely affect energy use. Alternatively, these issues generate energy losses, i.e. lower-quality inefficient appliances and the lack of technical possibilities of dwellings raise the energy expenditures of households and, in turn, energy bills. Thereby, energy-inefficient dwellings generate more expenditures rather than the energy-efficient dwellings, implying that lower-income households, which are unable to buy high-quality equipment, spend more money on energy services. Hence, poor housing conditions and the aforementioned structural issues affect the quality of households and the energy expenditures.

Given the income perspective, the economic conditions of states significantly affect the energy-poor. Lower-income households face several difficulties in affording modern energy services, which, in turn, affect their living conditions and well-being (Owusu et al., 2016). Alternatively, poor households are mostly deprived of monetary sources to cover energy needs, i.e. they are unable to afford cooling and heating services or even to cover daily mobility needs (Lucas et al., 2016). Nevertheless, energy poverty does not imply income poverty, i.e. these terms are not equivalent. Although energy and income poverty both reflect economic deprivation and poor living conditions, it is accepted that they measure different aspects of poverty (Thomson et al., 2019).

Currently, soaring energy prices have been one of the most significant drivers of energy poverty, and the energy market affairs have raised energy and economic disparities. The liberalisation, the geopolitical risks and the rivalry of energy market systems significantly affect the distribution, the aggregate demand and supply, the tariffs and the government intervention on energy resources and, consecutively, the outcomes of energy poverty (Trinomics, 2020). Hence, market rivalry, tariff-specific preferences and tariff choices may disturb the formation of energy prices, in favour of consumers’ protection (Dobbins et al., 2015). The energy price shock on the European economy has already deteriorated human well-being and economic welfare, while the expected consequences in the long run are destructive. According to the European Commission’s State of the Energy Union 2021, the steep increase in wholesale electricity prices, followed by rising gas prices, has caused detrimental effects on individuals. Verily, 31 million European citizens are considered as energy-poor in 2021, owing to energy prices spike. In the interim, the Russian invasion of Ukraine has caused a decay in global energy markets, i.e. an erratic surge in crude oil prices. Although the effects of domestic energy prices may vary due to country- and income-specific characteristics, they continue to be regressive. Ultimately, energy poverty is a multi-dimensional concept that needs to be acknowledged not only on the grounds of infrastructure, income and energy prices, but also from the political perspective.

## Conclusion

Energy poverty is an emerging issue towards global affairs. Currently, the shaping of energy-related policies is becoming essential, in the view of new societies. Given the Sustainable Development Goals, nations are recently bounded by uniform strategies and initiatives in favour of the energy-poor population. Overall, international energy agencies and governments seek to address energy-related issues that threaten sustainability. To that end, economies set energy policies that promote equity, social rights and inclusion. Howbeit, these policies may vary across countries, i.e. uniform policies do not sufficiently fit each county’s needs. In other terms, the effects of energy poverty between the European households significantly vary, a fact that is potentially attributed to country-specific, political-specific or geographical-specific characteristics. Hence, the understanding of the transitional patterns of energy poverty is essential for policymaking and decisions.

In this study, we investigate the dynamic patterns of energy poverty among 27 European economies between 2005 and 2020. We follow the study of Phillips and Sul (2007) to examine whether countries converge to an equilibrium steady state or converge to different steady states. The results of the data-driven algorithm suggest the existence of three convergence groups of the population unable to keep home adequately warm showcase. We consider that the affordability of heating services is adequately explained by structural conditions of housing, weather conditions (i.e. temperature outcomes) and energy costs. Besides, we find a greater number of convergence clubs in terms of the arrears on utility bills. The adverse financial conditions, followed by the Great Recession, have caused severe effects on employment and disposable income; hence, arrears on utility bills across Croatian, Romanian, Spanish, Bulgarian, Cypriot, Irish and Slovakian households are triggered. Economies such as Belgium, Sweden, Finland, Czechia and German prosper in terms of energy poverty, due to effective disconnection protection and income schemes, as well as building renovation schemes. Interestingly, the results of material deprivation on the housing dimension suggest the formation of two convergence clubs. We consider that a large part of the European population suffers severely from structural problems, i.e. the deficiency of basic sanitary facilities and other issues on infrastructural terms. Ultimately, we infer that a significant proportion of households struggle with energy-related issues that affect their living conditions and well-being. Several disconnection and income burdens significantly affect the distribution of energy across households and the share of population at risk of poverty. We suggest that the analysis of convergence hypothesis is a precondition of shaping effective energy-related policies between specific groups of the European countries.

Policymakers and international organizations of energy should orient future strategies towards buildings renovation and income schemes for the reconstruction of dwelling that lack basic sanitary facilities. Precisely, economies like Portugal, Italy, Bulgaria, Romania, Latvia, Hungary, Lithuania and Greece are among the EU members with the highest rates of severe housing deprivation. To that end, policymakers should adopt long-term measures to protect vulnerable consumers and guarantee their access to basic energy resources. The renovation of old buildings which have poor thermal characteristics or suffer from leaking roofs, lack of bath or toilet and damaged walls is essential to improve the efficiency in dwellings. The investments in green energy and the effective exploitation of innovative technologies in buildings renovation are fundamental mechanisms that can improve the efficient use of energy within households. The construction of sanitary facilities entails the access to energy for all households, eliminates the energy inequalities and protects the people who are at risk of poverty. In view of the building renovation, low-income households who are in significant arrears on utility bills will lower the average cost of energy and increase their disposable income. Hence, these measures are not income related, i.e. they do not provide budget support in households. Instead, they are long-term schemes which will potentially improve the residential use of energy and the energy efficiency in the household sector. So, we suggest that the buildings renovation for the aforementioned economies is essential for governments to improve the living standards of the vulnerable population.

However, it is significant to highlight the necessity for short-term measures which are income related. To be more precise, people who reside in countries or regions that face extreme climatic conditions, i.e. heat or cold waves, are hit hard during summer or winter periods. Nevertheless, this is mainly evident to poor households who lack thermal or cooling services. Verily, these waves have detrimental effects on human health and life expectancy. Southeast and Eastern European countries face hot and dry summer, which implies that individuals who are unable to afford for cooling services are at risk of severe health problems. Cyprus, Spain, Portugal, Bulgaria and Lithuania are the most vulnerable members which are hit by continental warm summer and cold winter, implying the emergent need for the governments’ action. Governments should grant heating or cooling allowances to vulnerable consumers or social tariffs for electricity. The provision of income support schemes is essential for the replacement of ineffective appliances or the access to cooling or heating services. Besides, the use of renewables and effective allowances not only protects socially vulnerable consumers, but it also improves the social welfare and sustainable development and mitigates the greenhouse gas emissions.

Moreover, we recommend that information and awareness measures towards the effective use of energy will potentially contribute to the energy resources services. Instead, energy inequalities may be misleading due to the variation or even the lack in the definitions of vulnerable consumers and energy poverty. Inconsistent results and data collection on energy poverty indicators adversely affect policy measures and the initiatives, as well as the population that receive support by governments. Hence, we infer that the European Member States should adopt uniform and flexible measures of energy poverty, to protect vulnerable consumers by means of the access and affordability of energy services and well-being. Conclusively, energy poverty alleviation is a profound dimension for transition and welfare economies.

Finally, a major limitation of this paper is the lack of the investigation of potential factors that drive energy poverty outcomes. The examination of these factors across convergence clubs would provide sufficient information in terms of shaping country-specific policies. Therefore, future research on the determinants of energy poverty convergence clubs could prove beneficial to the energy poverty research.

## Data Availability

Our manuscript contains data, which will be made available on reasonable request. Ethical approval and consent to participate The research carried out in this work was taken into account with respect to the observance of all the rules of ethics that govern the conduct of such research.

## Footnotes

1 Evaluation of the EU’s regulatory framework for electricity market design and consumer protection in the fields of electricity and gas (https://eur-lex.europa.eu/resource.html?uri=cellar:20674470-b7b9-11e6-9e3c-01aa75ed71a1.0001.02/DOC_1&format=PDF).
